# Developing and validating a prediction model of adolescent major depressive disorder in the offspring of depressed parents

**DOI:** 10.1111/jcpp.13704

**Published:** 2022-09-12

**Authors:** Alice Stephens, Judith Allardyce, Bryony Weavers, Jessica Lennon, Rhys Bevan Jones, Victoria Powell, Olga Eyre, Robert Potter, Valentina Escott Price, David Osborn, Anita Thapar, Stephan Collishaw, Ajay Thapar, Jon Heron, Frances Rice

**Affiliations:** ^1^ Wolfson Centre for Young People's Mental Health, Section of Child and Adolescent Psychiatry, Division of Psychological Medicine and Clinical Neurosciences, School of Medicine Cardiff University Cardiff UK; ^2^ MRC Centre for Neuropsychiatric Genetics and Genomics, Division of Psychological Medicine and Clinical Neurosciences, School of Medicine Cardiff University Cardiff UK; ^3^ Division of Psychiatry University College London London UK; ^4^ Camden and Islington NHS Foundation Trust London UK; ^5^ Centre for Academic Mental Health, Population Health Sciences, Bristol Medical School Bristol University Bristol UK

**Keywords:** Risk prediction, pre‐emptive, prevention, depressive disorder, ALSPAC

## Abstract

**Background:**

Parental depression is common and is a major risk factor for depression in adolescents. Early identification of adolescents at elevated risk of developing major depressive disorder (MDD) in this group could improve early access to preventive interventions.

**Methods:**

Using longitudinal data from 337 adolescents at high familial risk of depression, we developed a risk prediction model for adolescent MDD. The model was externally validated in an independent cohort of 1,384 adolescents at high familial risk. We assessed predictors at baseline and MDD at follow‐up (a median of 2–3 years later). We compared the risk prediction model to a simple comparison model based on screening for depressive symptoms. Decision curve analysis was used to identify which model‐predicted risk score thresholds were associated with the greatest clinical benefit.

**Results:**

The MDD risk prediction model discriminated between those adolescents who did and did not develop MDD in the development (*C*‐statistic = .783, IQR (interquartile range) = .779, .778) and the validation samples (*C*‐statistic = .722, IQR = −.694, .741). Calibration in the validation sample was good to excellent (calibration intercept = .011, *C*‐slope = .851). The MDD risk prediction model was superior to the simple comparison model where discrimination was no better than chance (*C*‐statistic = .544, IQR = .536, .572). Decision curve analysis found that the highest clinical utility was at the lowest risk score thresholds (0.01–0.05).

**Conclusions:**

The developed risk prediction model successfully discriminated adolescents who developed MDD from those who did not. In practice, this model could be further developed with user involvement into a tool to target individuals for low‐intensity, selective preventive intervention.

## Introduction

Major depressive disorder (MDD) is a leading global cause of lifelong disability. The incidence of MDD rises substantially during adolescence (Rohde, Lewinsohn, Klein, Seeley, & Gau, 2013), and first onset of depression at this age can predict a long‐term trajectory of symptoms into adulthood with poor long‐term mental health, social and educational outcomes (Rohde et al., 2013; Thapar, Collishaw, Pine, & Thapar, 2012). The most common major risk factor for adolescent MDD is depression in a parent. In the United Kingdom, approximately one in five children aged 0–16 years have a mother with a diagnosis of depression (Abel et al., 2019) and approximately one in three when considering offspring aged 0 to 30 years (Brophy, Todd, Rahman, Kennedy, & Rice, 2021). Depression in a parent increases the likelihood of depression in offspring by approximately three‐ to fourfold compared to healthy comparison groups (Rice, Harold, & Thapar, 2002) with this rising to 10‐fold when parental depression is severe (Weissman, 2016).

Multiple scientific and policy reports highlight the offspring of parents with mental illness, most commonly depression, as a group meriting special consideration for early identification of risk of depression and receiving preventive interventions (Weissman, 2016; World Health Organization, 2013). Indeed, there is increasing interest in targeted prevention to circumvent some of the poor outcomes seen in this group and effective programmes for preventing mood problems exist (Havinga et al., 2021). Nonetheless, the children of depressed parents represent a major missed opportunity for pre‐emptive psychosocial intervention because levels of identification of symptomatology and help seeking are low in this group (Potter et al., 2012). This is despite evidence showing that preventive interventions in offspring of depressed parents are effective in preventing depression onset and improving later functioning (Brent et al., 2015; Havinga et al., 2021; Loechner et al., 2018). However, not all offspring of depressed parents develop depression (Collishaw et al., 2016), and thus it is not necessarily efficient or cost‐effective to provide prevention to all. Risk prediction models to support the identification of young people at particular risk of developing MDD could help stratify this group and improve the early identification of those requiring preventive intervention, though we are not aware of any previous prediction models focusing on this group. Such a tool could involve using an individual's risk profile across a set of factors to guide clinical decision making and provide a rationale for providing prevention. Once risk prediction models have been derived, a widely used approach to evaluate the clinical consequences of using such a tool in practice is decision curve analysis which helps identify the levels of individual predicted risk where the clinical benefits of identifying elevated risk for depression outweigh any potential harms (Vickers, van Calster, & Steyerberg, 2019). Given that the clinical aim of such a tool would be to support access to preventive intervention and the existence of effective low‐risk preventive interventions (e.g. psychoeducation, self‐management strategies), we anticipated that lower thresholds of elevated risk would be informative for guiding clinical action (Havinga et al., 2021; NICE, 2019; Vickers et al., 2019).

In a longitudinal study of the adolescent offspring of depressed parents, we:

1. developed a risk prediction model to identify young people at the highest risk of developing MDD.
2. evaluated the performance of that prediction tool by:
  - comparing the performance of the MDD prediction model to a simple prediction model that included a depression screening questionnaire and age and sex as predictors.
  - testing the validation (transportability) of the risk prediction model in an independent sample.
  - using decision curve analysis to examine a range of appropriate clinical cut points (thresholds) for application of the risk prediction model in clinical practice.

## Methods

### Development sample

We developed our model in the Early Prediction of Adolescent Depression (EPAD) study – a prospective longitudinal study of the offspring (born between 1990 and 1998) of parents with recurrent depression described elsewhere (Mars et al., 2012; Rice et al., 2017; Appendix S1). In brief, 337 parents (315 mothers and 22 fathers) and their biological offspring [aged 9 to 17 (mean (SD) 12.4 (2.0) years); 197 girls and 140 boys] were recruited mainly from South Wales (United Kingdom) general practices. Recurrent depression in the index parent was defined as the presence of at least two episodes of *DSM‐IV* MDD (American Psychiatric Association, 2000). Data come from three assessment waves of parents and offspring (data collection between April 2007 and March 2011). The outcome was MDD at follow‐up defined as MDD at *either* the second or third assessment wave (median follow‐up from baseline = 2.5 years, range 1–4 years). Predictor variables were assessed at baseline (Wave 1). Written informed consent/assent was obtained from parents and children/adolescents as appropriate. The Multi‐Centre NHS Research Ethics Committee for Wales approved the study.

### Validation sample

Data were from a subsample of the Avon Longitudinal Study of Parents and Children (ALSPAC), an ongoing, longitudinal, population‐based UK cohort study. ALSPAC enrolled a sample of 14,541 pregnant women residing in Avon, England, with expected delivery dates between 1 April 1991 and 31 December 1992. Of these births, 13,988 children were alive at 1 year. In addition, 913 children were enrolled after age 7 years, giving a total sample of 14,901 children alive at 1 year (Boyd et al., 2013; Fraser et al., 2013). Note that the study website contains details of all the data that are available through a fully searchable data dictionary and variable search tool (http://www.bristol.ac.uk/alspac/researchers/our‐data/). We selected a subsample of offspring whose mothers self‐reported recurrent depression (*n* = 1,384). In line with previous work (Hammerton, Harold, Thapar, & Thapar, 2013), recurrent depression was defined as present when the mother reported two or more occasions of depression at least one of which was severe according to eight regular mother‐reported questionnaires collected between pregnancy and child age 12 years. The outcome was MDD at follow‐up defined as MDD at *either* age 13 or age 15 years (the median follow‐up was 3 years, range 1–4 years). Predictors were assessed at baseline (age 11 years). These time points were selected for comparability with the development sample. Ethical approval for the study was obtained from the ALSPAC Ethics and Law Committee and the Local Research Ethics Committees.

The primary outcome was MDD at 2.5‐ (development sample) or 3‐year follow‐up (validation sample) according to *DSM‐IV* (American Psychiatric Association, 2000) criteria. In the development sample, this was defined as new‐onset MDD at Wave 2 or 3 according to either parent or young person report on the Child and Adolescent Psychiatric Assessment (CAPA; Angold & Costello, 2000) with diagnoses confirmed by clinical consensus from two experienced psychiatrists (AT and RP). In the validation sample, MDD was defined according to either parent report (at age 13) or adolescent report (at age 15) on the Development and Wellbeing Assessment (DAWBA; Goodman, Ford, Richards, Gatward, & Meltzer, 2000).

Predictor variables were included based on clinical knowledge, past research literature and likely clinical usefulness. One particular piece of past research that we used to select predictors associated with the onset of adolescent depression was a theoretical model of depression onset (Rice et al., 2017) which included: (a) demographic variables (age and sex); (b) clinical antecedents (symptoms of anxiety, depression, irritability and behavioural problems); (c) indicators of socioeconomic adversity and stressful life events; (d) clinical severity/current parental depression and family loading for depression. Further details are included in Appendix S2, Tables S1–S5.

### Measures of selected predictor variables in the development and validation samples

#### Age

In EPAD, age at baseline was coded around the sample median ≤12 years (0) or ≥13 years (1). In ALSPAC, this was a constant as all participants were age 11 at baseline.

#### Sex

Biological sex according to parent or adolescent report was coded as male (0) or female (1).

#### Anxiety

In both samples, the emotional problems subscale of the Strength and Difficulties Questionnaire (SDQ; Goodman, 1997) assessed anxiety. In EPAD, information from informants (mother and child) was combined by using the highest rating per item from parent or the child. In ALSPAC, the mother was the informant.

#### Depressive symptoms

Adolescent self‐reported depressive symptoms at baseline were dichotomised around the validated clinical cut point (≥12; Thabrew, Stasiak, Bavin, Frampton, & Merry, 2018) of the short Mood and Feelings Questionnaire (sMFQ).

#### Low income

We defined low parent‐reported household income as <60% of the median sample income separately in each sample. Parent‐reported gross family income per annum was coded on a 7‐point scale in EPAD. Maternal‐reported gross family income per week was coded on a 10‐point scale in ALSPAC. In EPAD, <60% of the median sample income was <£20,000 per annum and in ALSPAC it was ≤£239 per week.

#### Stressful life events

In both datasets, we calculated a short screening measure of recent (past 12 months) negative stressful events with a possible range of 0 to 2 (coded 0, 1, 2+; Appendix S2, Table S1). This included the death of a family member and parental separation or parental discord. In EPAD, a life event was considered present if it was reported by the parent or the child; in ALSPAC, mothers reported on life events since child age 10 years. Steps to harmonise measurement across the two samples are described in Appendix S2.

#### Baseline parent depression

In EPAD, baseline index parent depression was defined using a cut point of ≥10 on the self‐reported Patient Health Questionnaire (PHQ‐9; Kroenke, Spitzer, & Williams, 2001). In ALSPAC, current maternal depression at baseline was defined (yes = 1: no = 0) using a cut point of ≥13 on the Edinburgh Postnatal Depression Scale (EPDS), (Cox, Holden, & Sagovsky, 1987).

### Statistical analysis

#### Model development

To arrive at the ‘MDD prediction model’, scientific and clinical judgements were used. We used logistic regression to predict MDD. As a comparison, a simpler prediction model including only depressive symptoms dichotomised around the clinical cut point, age and sex (the ‘simple comparison model’) was also estimated. We assessed the discrimination of those who did and did not develop MDD with the area under the curve (AUC; equivalent to the *C*‐statistic for binary outcomes) where a value of 0.5 represents chance and 1 represents perfect discrimination. Analyses were performed using Stata MP version 16.1. We carried out a formal sample size calculation using the method outline by Riley et al. (2020; Appendix S3) using the pmsampsize package in Stata. This showed that the development sample was powered to derive precise estimates (Table S6).

### Model validation: discrimination and calibration

Using the parameter estimates from model estimation in the development sample, we derived the predicted probability of MDD for each participant using the same set of predictor variables in the validation dataset. We assessed model performance using the *C*‐statistic (for discrimination) and calibration slope (C‐slope) and intercept (calibration in the large; CITL). Prevalence and measurement differences between samples can affect calibration in validation samples (Collins & Altman, 2012; Rocha et al., 2021). Recalibration updates to prediction models are recommended in such circumstances. We therefore followed the steps outlined in Steyerberg, Borsboom, van Houwelingen, Eijkemans, and Habbema (2004) for model recalibration. We updated (a) the calibration intercept and (b) both intercept and slope before (c) auditioning each individual predictor variable. In this final step, we investigated whether any predictor in the validation sample was associated with MDD once the linear predictor (predicted log odds of MDD) had been included in the logistic model. While recalibration steps (a) and (b) only affect calibration, the third step will also affect discrimination. This third step yielded a fully updated MDD prediction model which we considered, in addition to the original MDD prediction model, in terms of clinical utility.

### Clinical utility

We used decision curve analysis to assess the clinical usefulness of the prediction model by estimating net benefit. Net benefit is a metric of true positives minus false positives for a given threshold of predicted risk (Vickers et al., 2019; Vickers, Van Calster, & Steyerberg, 2016). We made the decision that lower thresholds of elevated risk between 1% and 15% (i.e. the predicted risk of MDD for an individual) were likely to be relevant thresholds for alerting clinical action. We drew a decision curve and compared this to a ‘treat‐all’ strategy (Vickers et al., 2016, 2019). We additionally calculated the positive predictive value (PPV), the negative predictive value (NPV), sensitivity, specificity and the prevalence of individuals at or above the risk thresholds of 5%, 10% and 15%.

To address missing data, we employed multiple imputation (MI) using a fully conditional specification (FCS) approach (–mi imputed chained– in Stata) which imputes each incomplete variable univariately using an appropriate regression model. One hundred imputed datasets were produced for the development and validation samples and 25 iterations were used within the FCS routine (Appendix S4).

## Results

Table 1 presents descriptive statistics for the predictor variables included in the MDD prediction model for both datasets. These show that in the development sample, elevated depressive and anxiety symptoms at baseline and MDD at follow‐up were more common, and the age range was substantially wider.

**Table 1:** Descriptive statistics of predictor and outcome variables in the discovery (EPAD) and validation (ALSPAC) samples

	EPAD (development sample)	ALSPAC (validation sample)
	*N* = 337	*N* = 1,384
Predictor variables	*N* (%) or mean (*SD*)	*N* (%) or mean (*SD*)
Female sex	197 (58.5%)	701 (50.7%)
Low income (% yes)	80 (28.5%)	218 (25.0%)
Anxiety score	3.01 (2.56)	2.05 (1.95)
Depression score above cut point (≥12)	54 (16.0%)	46 (3.3%)
Stressful life events (% endorsing one or more event)	82 (25.8%)	289 (29.0%)
Age (<12 years)	181 (53.7%)	1,384 (100%)
Age (13+ years)	156 (46.3%)	0 (0%)
Parent current depression (% yes)	123 (39.1%)	341 (34.3%)
Outcome variable		
MDD at follow‐up	26 (7.7%)	40 (4.7%)

In complete case analysis, amounts of missing data are as follows: In EPAD, the development sample: sex = 0; low income = 57; anxiety score = 19; depression score above cut point = 26; stressful life events = 19; age = 0; parent current depression = 22; MDD at follow‐up = 0. In ALSPAC, the validation sample: sex = 0, low income = 511, anxiety score = 508; depression score above cut point = 510; stressful life events = 391; age (constant) = 0; parent current depression = 391; MDD at follow‐up = 524.

Table 2 shows results of the full MDD and the simple comparison prediction models in the development dataset. Log odd estimates and intercepts from these models are shown in Table S7. Across the 100 imputed datasets, the MDD prediction model had a median *C*‐statistic of .783 [IQR (interquartile range): .779, .788] showing that it could discriminate between those who did and did not develop MDD at follow‐up in the development sample. The range of predicted probabilities was .006 to −.556 (IQR = .027, .097; Figure S1) illustrating that the model identified variation among study members in absolute levels of individual risk. In contrast, the simple comparison model (including age, sex and the short MFQ depression questionnaire) showed poorer discrimination with a median *C*‐statistic of .659 (IQR: .643–.661) and there was a lower range of predicted probabilities [.030 to .173 (IQR = .053, .117)] (Figure S2).

**Table 2:** Multivariable logistic regression models (MDD prediction model and simple comparison model) in discovery sample (EPAD)

Variable	OR	95% CI	*p*	*C*‐statistic if variable removed from model	*C*‐statistic
MDD prediction model					
Female sex	2.23	0.82, 6.08	.117	.764 (.760–.770)	.783 (.779–.788)
Age (13+ years)	2.04	0.85, 4.87	.109	.771 (.768–.775)	
Parental current depression	0.73	0.28, 1.85	.502	.783 (.780–.788)	
Anxiety	1.72	1.15, 2.58	.009	.725 (.717–.730)	
Low income	1.80	0.69, 4.69	.228	.769 (.767–.772)	
Stressful life events					
One event	2.35	0.87, 6.39	0.093	.759 (.755–.764)	
Two or more events	2.54	0.54, 12.00	.239		
Simple comparison model					
Female sex	2.36	0.92, 6.10	.075	.601 (.595–.603)	.659 (.643–.661)
Age (13+ years)	1.81	0.79, 4.15	.164	.614 (.606–.616)	
Depression score above cut point	1.36	0.52, 3.59	.534	.643 (.643–.643)	

Imputed data (*n* = 337) estimates pooled across 100 datasets. Note there is no variability in the estimate for depression score in the simple comparison model because there is no missing data if depression score is removed from the simple comparison model. For full reporting, the log odds estimates including the intercepts are included in Table S7. Number of participants: 337 for EPAD with 26 outcome events.

### Validation

In ALSPAC, the median *C*‐statistic was .698 (IQR: .675, .719) for the MDD prediction model. As in the development sample, the simple comparison model showed poorer discrimination with a median *C*‐statistic of .554 (IQR: .536, .572) indicating near‐chance discrimination. We therefore did not evaluate the simple comparison model further. We next considered calibration of the full prediction model. Calibration assesses agreement between predicted and observed probabilities with CITL plotting mean observed and predicted values [median CITL = .011 (IQR: −.058, .103)] and C‐slope the slope of observed and predicted values [median C‐slope = .851 (IQR: .739, .941)]. An excellent CITL value (near zero) indicated that the model does not systematically over‐ or underestimate risk of MDD at follow‐up. However, the C‐slope value of <1 indicated predicted estimates that were too extreme (Van Calster et al., 2019). We then followed the recalibration steps outlined in the Methods section. This indicated that the variable ‘low income’ performed differently in the development and validation datasets. The fully updated MDD prediction model therefore updated the calibration intercept and slope and accounted for this difference in the predictive performance of ‘low income’ in the two datasets. This fully updated MDD prediction model showed improved discrimination between those who did and did not develop MDD in the validation sample with a median *C*‐statistic of .722 (IQR = .694, .741).

### Clinical utility

**Figure 1:**
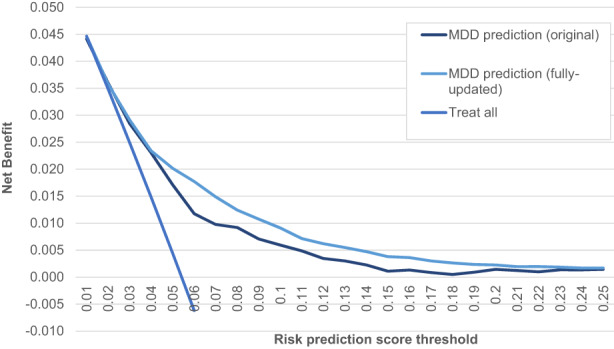
Net benefit of the full and fully updated MDD prediction models [Color figure can be viewed at wileyonlinelibrary.com]

Figure 1 presents a decision curve of the full and the fully updated (i.e. recalibrated) MDD prediction model. Figure 1 shows that the fully updated MDD prediction model has the highest net benefit across all risk thresholds we examined and that the benefit is higher at lower thresholds of risk (also see Table S8). We compared the decision curve for the MDD prediction model(s) to a ‘treat all’ comparison because an easily employable alternative approach would be to offer or signpost all offspring of depressed parents to resources or preventive intervention. Higher benefits were observed for the MDD prediction model(s).

Table 3 gives statistics for the development of MDD across several thresholds of model‐predicted risk. There is a trade‐off between the positive predicted value (PPV; proportion of individuals with scores at the threshold who developed MDD) and the sensitivity (the proportion of future MDD cases who had predicted risks at that threshold). At model predicted risks of .05, PPV is 10.2 but sensitivity is maximised (67.2%) and this identifies one third of the sample. In contrast, PPV is maximal (21.7) at a threshold of .15, but 20.5% of future MDD cases have model predicted risks at this threshold and less than 5% of the sample have predicted risks at this level or higher. A transparent reporting of a multivariable prediction model for individual prognosis or diagnosis (TRIPOD) checklist is available for this work (Appendix S5).

**Table 3:** Prediction statistics for MDD across differing risk thresholds of fully updated model‐predicted risk in the external validation sample

Threshold	Prevalence at or above threshold	PPV, median (IQR)	NPV, median (IQR)	Sensitivity, median (IQR)	Specificity, median (IQR)
.05	34.8 (31.9, 37.9)	10.2 (9.3, 11.3)	97.3 (96.9, 97.5)	67.2 (64.7, 70.5)	67.1 (63.7, 70.1)
.10	14.6 (11.7, 16.9)	15.5 (14.2, 16.5)	96.3 (95.9, 96.6)	40.1 (32.8, 45.9)	86.9 85.0, 89.6)
.15	4.9 (3.5, 7.2)	21.7 (19.6, 23.5)	95.6 (95.2, 95.9)	20.5 (14.4, 27.1)	95.9 (94.1, 97.1)

IQR, interquartile range.

## Discussion

In the adolescent offspring of depressed parents, we aimed to develop an individual risk prediction model that could be used to aid with the identification of young people at increased risk of depression for consideration for prevention and early intervention strategies. We focused on the adolescent children of depressed parents because this group represents a major lost opportunity for early intervention (Potter et al., 2012; Weissman, 2016) and because prevention in this group is effective (Havinga et al., 2021). Tools to support early identification of risk and signpost to resources and interventions in this group have the potential to interrupt the cycle of intergenerational transmission of depression and mitigate some of the poor outcomes seen in young people who experience depression early in life (Brent et al., 2015).

We developed a risk prediction model that was able to differentiate young people at familial risk who later went on to develop MDD over a median 2.5‐ to 3‐year follow‐up period (range 1– 4). This model was able to distinguish cases and controls in the development sample and in an independent validation sample at levels of discrimination similar to those reported in other areas of medicine and recommended for clinical use (e.g. conversion to psychosis from a high‐risk clinical state, Fusar‐Poli et al., 2019). The distribution of predicted risk scores showed that individuals at high familial risk varied widely in their absolute levels of risk, consistent with other research (Collishaw et al., 2016). The MDD prediction model included information on predictors that assessed individual, family and contextual factors. The discriminative ability of this model was markedly better than a simple comparison model including only a depression screening questionnaire, age and sex as predictors. The latter simple model performed no better than chance in the validation sample.

The potential of prediction models for improving health depends on their clinical utility and on a thorough consideration of how they can be implemented in clinical practice as well as on the availability of effective preventive and early intervention strategies. This involves not only characteristics of the predictive model such as discrimination and calibration but also on deciding the appropriate risk threshold for the development of future MDD that leads to action by the clinician. We considered lower thresholds of absolute levels of risk as potential points for action by clinicians due to the availability of low‐risk, cost‐effective interventions to support mental health in young people at risk of developing MDD. These interventions include psychoeducation and training in coping skills, cognitive reappraisal, behavioural activation and lifestyle changes (Havinga et al., 2021). Results from decision curve analysis and from evaluation of sensitivity and NPV (Table 3; Table S8) confirmed the benefit of applying the model was greatest at relatively low risk score thresholds (between .01% and .05%).

Strengths of the study involve the use of well‐characterised, longitudinal, independent development and validation samples. Limitations include a relatively small development sample and low rates of MDD incidence since we used gold standard measures to define MDD in a relatively young population of adolescents. We chose to focus on adolescent‐onset MDD given the need for improving early intervention because of the poor prognosis when depression starts early (Weavers et al., 2021). In the development sample, formal power calculations showed that the sample size was sufficient to derive precise estimates despite the relatively low incidence of MDD. However, the low incidence of MDD in the validation sample means that the external validation study is likely under‐powered for testing whether the developed prediction model derived accurate and precise estimates of absolute risk in an independent sample. Nonetheless, it is recognised that too many models are discarded following single nonvalidations which wastes previous effort and that there is a need for multiple independent validation studies before deciding whether a risk model would be useful to implement in practice (Collins, Ogundimu, & Altman, 2016). Indeed, we are aware of only one previous study focusing on adolescent depression risk prediction which did not find evidence for transportability of estimates as the model did not validate across independent datasets (Rocha et al., 2021). This further highlights the importance of future work to externally validate adolescent depression prediction models across multiple datasets. Furthermore, the risk prediction model developed here would need careful consideration of how it could be implemented in ways that are consistent with the needs of young people, families/carers and clinicians, therefore future work would also need to consider practical issues around implementation. For instance, stigma in families where a parent has a mental illness is often associated with concealment of the mental health problem which then leads to a lack of help seeking in family members (Reupert et al., 2021). Also, there is a growing emphasis on providing early mental health intervention and prevention programmes in schools (Shinde et al., 2018). However, schools, unlike general practitioners, do not have access to information on parent mental health. There is therefore clearly a need to work with relevant stakeholders to understand how best to codesign and implement a risk prediction tool in a way that is consistent with and sensitive to their needs and does not unintentionally stigmatise or increase anxiety in young people or their parents (Appendix S6). There is also a need to consult with clinicians to better understand how a tool might be implemented in clinical practice and how they might access interventions that are provided in another setting. A final consideration is the availability of interventions because access to psychological therapies is challenging outside of specialist services. However, many low‐intensity interventions are likely to be useful in the context of elevated risk to MDD in this group including increasing parental knowledge about when to seek help for their children's mental health, psychoeducation and self‐management strategies including activity scheduling and healthy sleep, diet and exercise routines (Bevan Jones et al., 2018; Havinga et al., 2021).

In summary, we present a model for a prediction tool that can discriminate young people at heightened familial risk of depression who go on to develop MDD. This tool is potentially useful for improving the access of this group to early support and intervention including psychoeducation and self‐management strategies. The use of such tools in routine clinical practice could be one part of the armamentarium to mitigate the intergenerational cycle of depression in families by helping to implement scientific knowledge into practice. Such tools will likely require further development including work with stakeholders.

## Supporting information


**Appendix S1.** Additional details of development sample.
**Appendix S2.** Details on the selection process for predictor variables considered for inclusion in MDD prediction model.
**Appendix S3.** Sample size estimation.
**Appendix S4.** Missing data considerations and multiple imputation.
**Appendix S5.** TRIPOD checklist for prediction model development.
**Appendix S6.** Information on our initial public, patient and involvement (PPI) work.
**Table S1.** Predictor variables considered for inclusion in MDD prediction model.
**Table S2.** Predictor variables included in simple comparison model.
**Table S3.** Univariable association with MDD at follow‐up of original predictor variables in the discovery sample (EPAD) in observed data.
**Table S4.** Backwards stepwise logistic regression on all variables to identify strongest predictors.
**Table S5.** Univariable association with MDD at follow‐up of final predictor variables in the discovery sample (EPAD) in observed data.
**Table S6.** Sample size estimation results.
**Table S7.** Log odds estimates including intercepts for the full and simplified MDD prediction model in the development sample.
**Table S8.** Net benefit figures for range of prediction models in the validation sample.
**Figure S1.** Range of predicted probabilities from the MDD prediction model.
**Figure S2.** Range of predicted probabilities from the simple comparison prediction model.
