# Supplementary material for: Developing and validating a prediction model of adolescent major depressive disorder in the offspring of depressed parents
Source: J Child Psychol Psychiatry. 2022 Sep 12;64(3):367–75. doi: 10.1111/jcpp.13704 (PMC10087673; doi:10.1111/jcpp.13704)
Supplement: Supplementary file 1 — Appendix S1. Additional details of development sample. Appendix S2. Details on the selection process for predictor variables considered for inclusion in MDD prediction model. Appendix S3. Sample size estimation. Appendix S4. Missing data considerations and multiple imputation. Appendix S5. TRIPOD checklist for prediction model development. Appendix S6. Information on our initial public, patient and involvement (PPI) work. Table S1. Predictor variables considered for inclusion in MDD prediction model. Table S2. Predictor variables included in simple comparison model. Table S3. Univariable association with MDD at follow‐up of original predictor variables in the discovery sample (EPAD) in observed data. Table S4. Backwards stepwise logistic regression on all variables to identify strongest predictors. Table S5. Univariable association with MDD at follow‐up of final predictor variables in the discovery sample (EPAD) in observed data. Table S6. Sample size estimation results. Table S7. Log odds estimates including intercepts for the full and simplified MDD prediction model in the development sample. Table S8. Net benefit figures for range of prediction models in the validation sample. Figure S1. Range of predicted probabilities from the MDD prediction model. Figure S2. Range of predicted probabilities from the simple comparison prediction model. [file JCPP-64-367-s001.docx]

**Developing and validating a prediction model of adolescent major depressive disorder in the offspring of depressed parents**

**Supporting Information**

**Appendix S1.** Additional details of development sample

**Appendix S2.** Details on the selection process for predictor variables considered for inclusion in MDD prediction model.

**Table S1.** Predictor variables considered for inclusion in MDD prediction model.

**Table S2.** Predictor variables included in simple comparison model.

**Table S3.** Univariable association with MDD at follow-up of original predictor variables in the discovery sample (EPAD) in observed data.

**Table S4.** Backwards stepwise logistic regression on all variables to identify strongest predictors.

**Table S5.** Univariable association with MDD at follow-up of final predictor variables in the discovery sample (EPAD) in observed data.

**Appendix S3.** Sample size estimation.

**Table S6.** Sample size estimation results.

**Appendix S4.** Missing data considerations and multiple imputation.

**Table S7** Log odds estimates including intercepts for the full and simplified MDD prediction model in the development sample.

**Figure S1.** Range of predicted probabilities from the MDD prediction model.

**Figure S2.** Range of predicted probabilities from the simple comparison prediction model

**Table S8.** Net benefit figures for range of prediction models in the validation sample.

**Appendix S5.** TRIPOD checklist for prediction model development.

**Appendix S6.** Information on our initial public, patient and involvement (PPI) work.

**References.**

**Appendix S1. Additional details on the development sample**

Recurrent depression in the index parent was confirmed at baseline as the presence of at least two episodes of DSM-IV major depressive disorder (MDD) using a timeline of the parent’s depressive episodes (Belli, 1998) and the Schedules for Clinical Assessment in Neuropsychiatry (Wing et al., 1990) assessed current parental depression. One child per family was included. All children were living with the affected parent. Additional exclusion criteria were moderate to severe intellectual disability (IQ, <50) in the child and the presence of DSM-IV criteria for bipolar disorder, mania or hypomania, or psychotic disorder in the parent at baseline interview. Data come from three assessments of parents and offspring. Offspring were born between 1990 and 1998 and this paper presents data collected from three assessments where data were collected during 2007 (baseline, T1), 2008/9 (second assessment, T2) and 2009/10 (third assessment, T3). The mean (SD) time between the baseline (T1) and second (T2) assessment was 16.2 (2.6) months and between the second and third (T3) assessment was 12.5 (1.6) months. Data were collected via semi-structured diagnostic interviews and questionnaires (Mars et al., 2012).

**Appendix S2. Details on selection of predictor variables**

Our starting point was a published theoretical model of adolescent depression onset (Rice et al., 2017). This model tested whether multiple indicators of familial risk and social adversity predicted adolescent onset MDD via related dimensional clinical antecedents. It found direct effects of indicators of family risk (parental severe depression, family history of depression in additional 1^st^ and 2^nd^ degree relatives), social adversity (economic disadvantage, recent stressful life events) and the dimensional clinical antecedents of irritability and anxiety on adolescent onset depression. That published model therefore found evidence that six factors predicted the onset of adolescent depression. We made the decision to additionally include age and sex in the prediction model given the strong associations of age and sex with adolescent MDD in the literature (Avenevoli et al., 2015). Table S1 outlines the predictor variables considered for inclusion in the prediction model. Our consideration of predictor variables proceeded in the following steps. First, we examined associations with MDD for all predictors originally considered in the published model plus age and sex (10 predictors). As we expected and consistent with the previously published work, this identified that not all predictors considered in the original theoretical model were important in predicting the outcome (Table 3). Next, we identified the strongest predictors by running a stepwise model (Table 4). This identified age, anxiety and stressful life events as showing the strongest associations with MDD. In the next step, we considered what theoretically important variables would be omitted if we selected only the three strongest predictors. We based these decisions on scientific evidence but also, when selecting a particular variable, we also considered the feasibility of assessing a particular measure in clinical practice. We elected to include age, sex, anxiety, stressful life events, poverty and parental depression given the strong evidence that these factors are associated with adolescent MDD (Avenevoli et al., 2015; Gunlicks & Weissman, 2008; Mars et al., 2012; Rice et al., 2017; Ridley et al., 2020; Wickramaratne et al., 2011). For parental depression, we selected current parental depression status rather than past severity or family history in other family members as we judged this variable would be easier and quicker to assess in clinical practice. Finally, once we had identified the key variables to include in the prediction model, we considered the feasibility of collecting information on these measures in a clinical setting and we selected shorter, simpler measures of the same construct where they were available. A final consideration in this last part of identifying the variables to include in the prediction modelling was the extent to which similar measures were available in the development and the validation data sets. This final issue about comparability of measurement across development and validation data sets was only relevant for the measurement of stressful life events which needed to be simplified to ensure harmonized measures across the two data sets (see later for details).

Once we had arrived at our selected pool of six predictors (sex, age, anxiety, stressful life events, low income, degree of familial risk for depression), we selected shorter measures of similar constructs when they were available. Specifically, for anxiety we selected the measure of the emotional problems subscale of the SDQ (5 items) rather than the SCARED (41 items), and for assessing current parental depression we selected a variable based on a screening questionnaire rather than on a clinical interview (Table S1). Finally, we considered the need for measurement of key concepts to be similar in the discovery and the validation (Van Calster et al., 2014) and amended the way variables were coded in the development sample where required. This applied to the assessment of stressful life events which were assessed differently in the development and validation data sets. To harmonize the way that stressful life events were measured in the predictor included in both data sets, we selected items that were overtly negative and were assessed in similar ways in both data sets. This included items in the categories “death of a family member” and “parental separation or parental discord” in each sample. In EPAD, the category death of a family member (yes/no) included the item “death of parent/brother/sister” and parental separation or parental discord (yes/no) comprised the terms: “parents divorced”, “parents quarrelled more” and “parent went to prison”. A life event was considered present if it was reported by the parent or the child (Gest et al., 1999). In ALSPAC, a short mother-reported screening measure of recent stressful life events (since child age 10 years) was calculated. This included death of a family member (yes/no) i.e., “child sibling died", and parental separation or parental discord (yes/no) i.e., “mother divorced", “mother argued with partner" and “mother convicted of offence". We therefore selected for inclusion, a stressful life events predictor coded (0, 1, 2+) that included 4-items that were assessed in both data sets.

Details on predictor variables included in the simple comparison model are outlined in Table S2. These were: age, sex and being above the published clinical cut-point of 12 on the self-reported shortened version of the Mood and Feelings Questionnaire which is a validated depression screening questionnaire for children and young people (Costello & Angold, 1988; Thabrew et al., 2018).

**Table S1**

**Selection process for predictor variables considered for inclusion in MDD prediction model**

| **Category of predictor variable considered for inclusion** | **Scale or measures available of this category in development sample** | **Measure selected for inclusion in final prediction model** | **If measure selected for inclusion in final prediction model differed from measure used in published theoretical model, what is the justification for this?** |
| --- | --- | --- | --- |
| 1. Sex | Female sex * | Sex | N/A |
| 2. Age | Age (<13 years or >= 13 years) * | Age<13 or >=13 | N/A |
| 3. Anxiety | SCARED *  SDQ emotional problems | SDQ emotional problem scale | Selected shorter measure |
| 4. Stressful life events | Life event screener *  Perceived stress scale ^a^ | 4-item stressful life event screener assessing the categories “death of a family member” and “parental separation  or discord” | Selected shorter measure to ensure similarity in measurement across development and validation data sets. |
| 5. Low income | Family income <=60% median in sample *  Struggling to make ends meet ^a^ | Family income <=60% median in sample | N/A |
| 6. Familial risk for depression | Past parental severe MDD episode with moderate-severe impairment (GAF<=50) or hospitalisation *  Weighted family history of depression *  Current probable parental depressive episode (parent PHQ-9 >10) | Current parent PHQ-9 score > >10) | Selected current parental depressive symptoms above clinical cut-point on screening questionnaire. Selected as short and simple to assess in clinical settings. |

Footnote to Table S1: * = original items considered as predictor variables as per Rice et al (2017). A = this was considered as a shorter alternative indicator of this predictor variable but was judged not to be comparable. MFQ = Mood and Feelings Questionnaire (Costello & Angold, 1988). SCARED = Screen for Child Anxiety Related Mood Disorders (Birmaher et al., 1999) SDQ = Strength and Difficulties Questionnaire (Goodman, 1997). PHQ-9 = Patient Health Questionnaire (Kroenke, Spitzer, & Williams, 2001). CAPA = Child and Adolescent Psychiatric Assessment. EPDS = Edinburgh Postnatal Depression Scale (Cox, Holden, & Sagovsky, 1987).

**Table S2**

**Details on predictor variables included in the simple comparison model**

| **Category of predictor variable considered for inclusion** | **Scale or measures available of this category in development sample** | **Measure selected for inclusion in final prediction model** | **If measure selected for inclusion in final prediction model differed from measure used in published theoretical model, what is the justification for this?** |
| --- | --- | --- | --- |
| 1. Sex | Female sex * | Sex | N/A |
| 2. Age | Age (<13 years or >= 13 years) * | Age<13 or >=13 | N/A |
| 3. Depression/ low mood | Mood and feelings questionnaire (long version) *  Mood and feelings questionnaire (short version)  In the past month, how often have you found that you could not cope? ^a^ | Short MFQ score clinical cut-point | Selected shorter depression measure |

Footnote to Table S2: A = this was considered as a shorter alternative indicator of this predictor variable but was judged not to be comparable. MFQ = Mood and Feelings Questionnaire (Costello & Angold, 1988).

**Table S3. Univariable association with MDD at follow-up of original predictor variables in the discovery sample (EPAD) in observed data.**

|  | **OR (95% CI)** | **P value** |
| --- | --- | --- |
| **Demographic variables** | **N=337** |  |
| Female sex | 2.52 (0.99-6.46) | 0.053 |
| Age group (< or => 13 years) | 1.95 (0.86-4.44) | 0.110 |
| **Clinical antecedents** |  |  |
| Child anxiety symptoms (SCARED) | 2.32 (1.50-3.59) | <0.001 |
| Child irritability symptoms (CAPA) | 1.58 (0.99-2.50) | 0.054 |
| Child depressive symptoms (long MFQ) | 2.01 (1.30-3.10) | 0.002 |
| Behaviour dysregulation | 1.23 (0.82 -1.84) | 0.311 |
| **Adversity** |  |  |
| Low income | 2.18 (0.87-5.48) | 0.098 |
| Stressful events | 1.82 (1.22-2.73) | 0.004 |
| **Clinical severity of parental depression including family history** |  |  |
| Parental severe depression (GAF score <50 and/or hospitalised) | 2.86 (0.82-10.01) | 0.100 |
| Family history weighted score | 1.33 (0.87-2.03) | 0.186 |

Footnote to Table S3: Analyses were completed using complete case data. SCARED = Screen for Child Anxiety Related Disorders; SDQ = Strength and Difficulties Questionnaire; CAPA – Child and Adolescent Psychiatric Assessment; MFQ= Mood and Feelings Questionnaire; PHQ = Patient Health Questionnaire; GAF = Global Assessment of Functioning (Hall & Parks, 1995). Analyses presented are a series of univariable analyses series of complete case data. Continuous predictor variables are standardized so odds ratios represent the change in odds of MDD for a 1 standard deviation unit increase in the predictor variable. Low income is a binary predictor variable.

**Table S4. Backwards stepwise logistic regression on all variables to identify strongest predictors.**

| Variable | Beta Coefficient | SE | P value | (95% CI) | C Statistic (95%CI) |
| --- | --- | --- | --- | --- | --- |
| Life events | 0.08 | 0.04 | 0.055 | (-0.00 – 0.16) | 0.82  (0.74-0.90) |
| Anxiety | 0.004 | 0.00 | <0.001 | (0.002 – 0.007) |  |
| Age (<13, =>13) | 0.10 | 0.06 | 0.005 | (0.03 – 0.16) |  |

**Table S5. Univariable association with MDD at follow-up of final predictor variables in the discovery sample (EPAD) in observed data.**

|  | **OR (95% CI)** | **P value** |
| --- | --- | --- |
| **Demographic variables** | **N=337** |  |
| Female sex | 2.52 (0.99-6.46) | 0.053 |
| Age group (< or => 13 years) | 1.95 (0.86-4.44) | 0.110 |
| **Clinical antecedents** |  |  |
| Child anxiety symptoms (SDQ) | 1.87 (1.29-2.71) | 0.001 |
| **Adversity** |  |  |
| Low income | 2.18 (0.87-5.48) | 0.098 |
| Stressful events | 2.28(1.00-5.20) | 0.049 |
| **Clinical severity of parental depression including family history** |  |  |
| Parental severe depression (PHQ) | 1.37(0.61-3.08) | 0.440 |

Footnote to Table S5 Analyses were completed using complete case data. Patient Health Questionnaire; SDQ (Strength and Difficulties Questionnaire. Analyses presented are a series of univariable analyses series of complete case data. Continuous predictor variables are standardized so odds ratios represent the change in odds of MDD for a 1 standard deviation unit increase in the predictor variable. Low income is a binary predictor variable

**Appendix S3. Sample size estimation**

Power analyses for prediction modelling in the development dataset was carried out according to the steps outlined in Riley et al (2020). This involves three steps with the final recommended sample size taken as the largest of these three calculations.

a) what sample size will produce a precise estimate of the overall risk outcome (aiming for a margin of error of <=.05 in the overall proportion estimate)?

b) what sample size will produce predicted values that have a small mean error (.05) across all individuals? I.e. a small absolute difference in the algorithm’s apparent and adjusted Nagelkerke’s R-squared value.

c) what sample size will produce a small required shrinkage of predictor effects (10% or less)?

We used the command psampsize in STATA to estimate the sample size calculations. We made the following assumptions:

1) We assumed an outcome prevalence of 8% based on estimates of adolescent MDD reported in high-risk samples (Rice et al., 2017).

2) We assumed an R^2^ of .20 based on a previous study finding that 17% of the variance of DSM-IV depressive symptom count could be explained by the six predicted variables (Rice et al., 2017) and on a previous risk prediction algorithm (Rocha et al., 2021). For the simple predictor model, we assumed a conservative R^2^ of .15.

3) As recommended by Riley et al 2020, we assumed shrinkage of <= 9%.

Table S5 shows results of sample size calculations based on these criteria

**Table S6. Sample size estimation results**

| Criteria | Sample size | Shrinkage | Parameters | R^2^ | Events per predictor |
| --- | --- | --- | --- | --- | --- |
| Full model | | | | | |
| Criteria 1 | 279 | .9 | 7 | .2 | 3.19 |
| Criteria 2 | 290 | .9 | 7 | .2 | 3.31 |
| Criteria 3 | 114 | .9 | 7 | .2 | 1.30 |
| Simplified model | | | | | |
| Criteria 1 | 165 | .9 | 3 | .15 | 4.4 |
| Criteria 2 | 128 | .9 | 3 | .15 | 3.41 |
| Criteria 3 | 114 | .9 | 3 | .15 | 3.04 |

Different approaches to estimating power for external validation are required (Riley et al., 2020). External validation primarily concerns the accurate (unbiased and precise) estimation of prediction model performance measures. For external validation, 100 events (i.e. affected cases) has been suggested as required but it is also important to note that multiple independent external validation studies are required before making decisions about the potential usefulness of a model. It has also been highlighted as important that developed models are not discounted based on a model not validating well in a single independent sample (Collins, Ogundimu, Altman, 2016).

**Appendix S4. Missing data considerations and multiple imputation**

To address missing data, we employed Multiple Imputation (MI) using a Fully Conditional Specification (FCS) approach (-mi imputed chained- in Stata) which imputes each incomplete variable univariately using an appropriate regression model. One hundred imputed datasets were produced for the development and validation samples and 25 iterations were used within the FCS routine. The information selected to include in the MI model included the variables included in the model, data related to model variables and predictive of dropout including earlier or proxy measures of the variables included in the model. Given the use of imputed data the following decisions were made. First, in the model development step, model building was carried out using only observed data. Second, the parameter values from the development model which were transported into the validation sample were pooled using Rubin’s rules (i.e., averaged across 100 datasets). Third, we take the approach used in Gerdin et al., (2016) and report all model performance statistics (discrimination, calibration, net benefit, PPV etc.) as the median value across the 100 imputed datasets along with the interquartile range (IQR). The validity of MI is dependent on the missing at random (MAR) assumption, namely that sufficient auxiliary information is available such that each incomplete variable can be rendered conditionally independent of its missingness; in other words, there remains no systematic difference between those with complete and non-complete data.

**Table S7. Log odds estimates including intercepts for the full and simplified model in the development sample.**

| Variable | Log odds | SE | 95% CI | P value |
| --- | --- | --- | --- | --- |
| **MDD prediction model** |  |  |  |  |
| Intercept | -3.931 | .599 | -5.11, -2.76 | .001 |
| Female sex | .801 | .511 | -.20, 1.80 | 0.117 |
| Age (13 + years) | .711 | .445 | -.16, 1.58 | 0.109 |
| Parental current depression | -.321 | .478 | -1.26, .62 | 0.502 |
| Anxiety | .542 | .208 | .14, .95 | 0.009 |
| Low income | .589 | .488 | -.37, 1.54 | 0.228 |
| Stressful life events |  |  |  |  |
| 1 event | .856 | .510 | -.14, 1.85 | 0.093 |
| 2 or more events | .933 | .792 | -.62, 2.49 | .239 |
| **Simple comparison model** |  |  |  |  |
| Intercept | -.344 | .484 | -4.39, -2.49 | .001 |
| Female sex | .860 | .483 | -.09, 1.81 | 0.075 |
| Age (13 + years) | .591 | .425 | -.24, 1.42 | 0.164 |
| Depression score above cut-point | .308 | .495 | -.66, 1.27 | 0.534 |

**Figure S1**

**Range of predicted probabilities from the MDD prediction model**

**
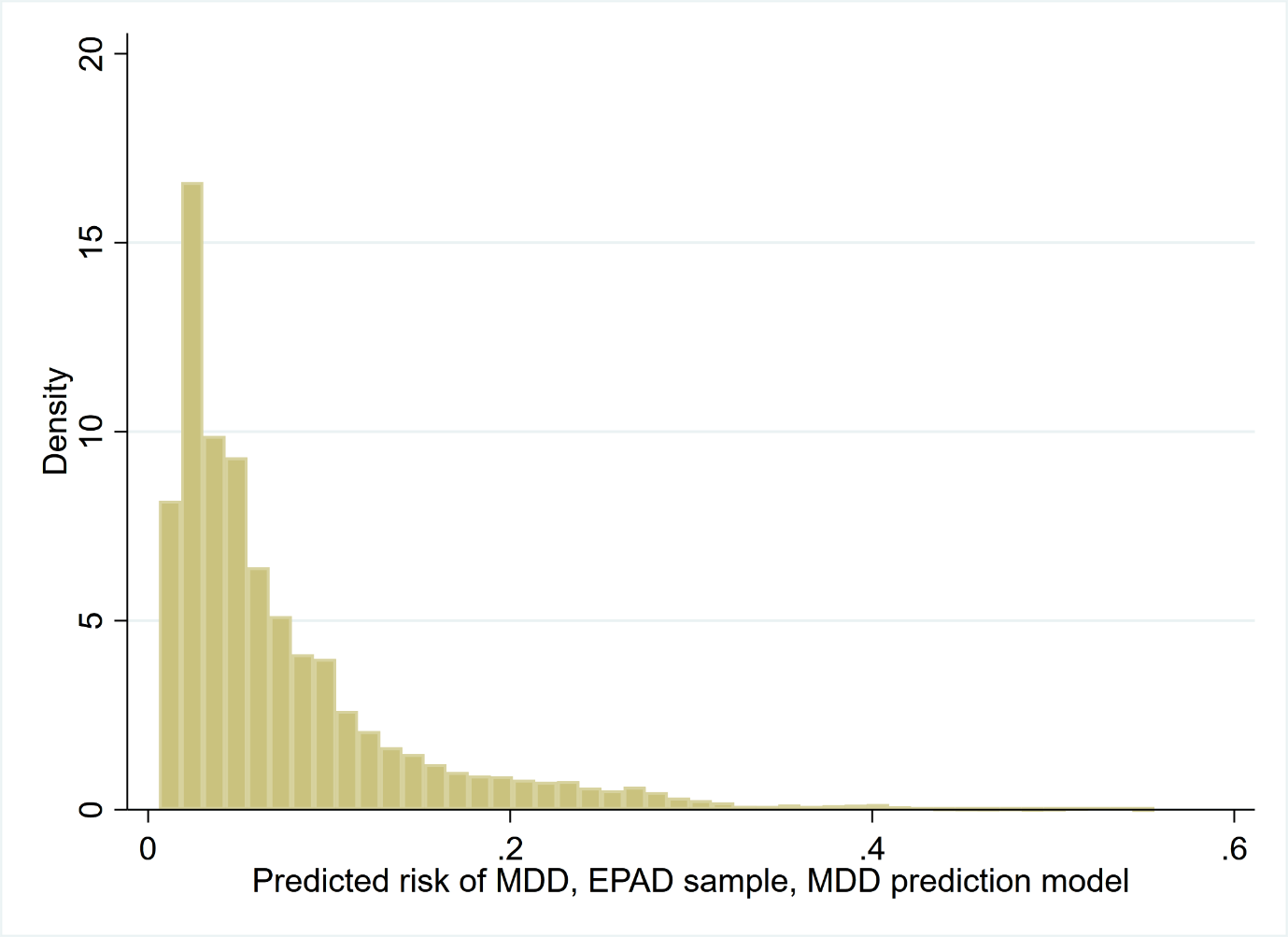
**

Footnote to Figure S1: Density = Number of participants

**Figure S2**

**Range of predicted probabilities from the simple comparison prediction model**


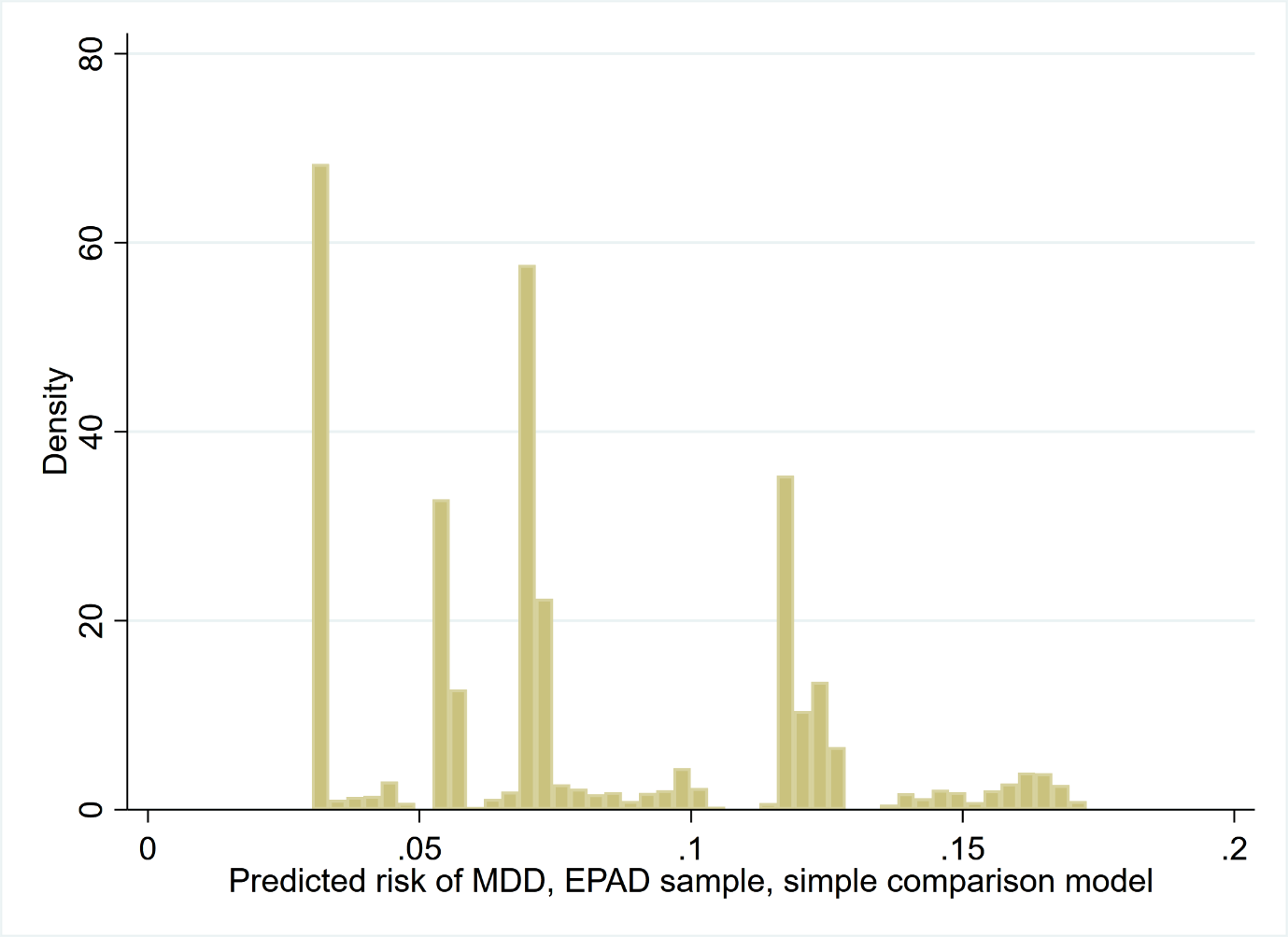


Footnote to Figure S2: Density = Number of participants

**Table S8.**

**Net benefit figures for range of prediction models in the validation sample**

| **Threshold** | **Net benefit**  **Treat all**  **Mean**  **(IQR)** | **Net benefit**  **Simple comparison model**  **Mean**  **(IQR)** | **Net benefit**  **MDD prediction model (original)**  **Mean**  **(IQR)** | **Net benefit**  **MDD prediction model (fully-updated)**  **Mean**  **(IQR)** |
| --- | --- | --- | --- | --- |
| .05 | .005  (.004, .009) | .005  (.002, .008) | .02  (.01, .02) | .02  (.02, .02) |
| .10 | -.05  (-.05, -.05) | .000  (.000, .000) | .006  (.004, .008) | .009  (.004, .008) |
| .15 | -.011  (-.12, -.11) | .000  (.000, .000) | .001  (-.001, .003) | .001  (.006, .01) |

**Appendix S5. TRIPOD Checklist: Prediction Model Development and Validation**

| **Section/Topic** | Item |  | Checklist item | Page |
| --- | --- | --- | --- | --- |
| **Title and abstract** |  |  |  |  |
| Title | 1 | D, V | Identify the study as developing and/or validating a multivariable prediction model, the target population, and the outcome to be predicted | P1 |
| Abstract | 2 | D, V | Provide a summary of objectives, study design, setting, participants, sample size, predictors, outcome, statistical analysis, results and conclusions | P2 |
| Introduction |  |  |  |  |
| Background and objectives | 3a | D, V | Explain the medical context (including whether diagnostic or prognostic) and rational for developing or validating the multivariable prediction model, including references to existing models | P4-P5 |
|  | 3b | D, V | Specify the objectives, including whether the study described the development of validation of the model or both. | P5 (study aims) |
| Methods |  |  |  |  |
| Source of data | 4a | D, V | Describe the study design or source of data (e.g. randomized trial, cohort, or registry data), separately for the development and validation data sets, if applicable. | P5-P7 with additional details on development sample in Supporting Information |
|  | 4b | D, V | Specify the key study dates, including start of accrual; end of accrual; and if applicable, end of follow-up. | P6 with additional details in Supporting Information |
| Participants | 5a | D, V | Specify key elements of the study setting (e.g. primary care, secondary care, general population) including number and location of centres. | Two cohort studies as described on P6 |
|  | 5b | D, V | Describe eligibility criteria for participants | P6-7 with additional details in Supporting Information |
|  | 5c | D, V | Give details of treatments received, if relevant | N/A |
| Outcome | 6a | D, V | Clearly define the outcome that is predicted by the prediction model, including how and when assessed. | P7 |
|  | 6b | D, V | Report any actions to blind assessment of the outcome to be predicted. | In development sample, parent and child interviewed separately, P7 |
| Predictors | 7a | D, V | Clearly define all predictors used in developing or validating the multivariable prediction model, including how and when they were measured. | Y, P8-9 with additional details in Supporting Information |
|  | 7b | D, V | Report any actions to blind assessment of predictors for the outcome and other predictors. | D & V - Questionnaires assessing predictors completed separately from clinical assessments of outcome |
| Sample size | 8 | D, V | Explain how the study size was arrived at. | D & V – both cohort studies. D - P6 includes formal power calculation results on P10 and full details in Supporting Information Table S5. V – P6 and P7 outline the inclusion criteria |
| Missing data | 9 | D, V | Describe how missing data were handled (e.g., complete-case analysis, single imputation, multiple imputation) with details of any imputation method. | P11 with further details in Supporting Information Appendix S3 |
| Statistical analysis methods | 10a | D | Describe how predictors were handled in the analyses. | P10-11 |
|  | 10b | D | Specify type of model, all model-building procedures (including any predictor selection), and method for internal validation. | P10 |
|  | 10c | V | For validation, describe how the predictions were calculated | P10 |
|  | 10d | D, V | Specify all measures used to assess model performance and, if relevant, to compare multiple models. | P10-11 |
|  | 10e | V | Describe any model updating (e.g. recalibration) arising from the validation, if done. | P10-11 |
| Risk groups | 11 | D, V | Provide details on how risk groups were created, if done. | V - P6-7. |
| Development vs validation | 12 | V | For validation, identify any differences from the development data in setting, eligibility criteria, outcome and predictors. | Table 1 P26 |
| Results |  |  |  |  |
| Participants | 13a | D, V | Describe the flow of participants through the study, including the number of participants with and without the outcome and, if applicable, a summary of the follow-up time. A diagram may be helpful. | Table 1 P26 |
|  | 13b | D, V | Describe the characteristics of the participants (basic demographics, clinical features, available predictors), including the number of participants with missing data for predictors and outcome. | Table 1 P26  Also, multiple imputation used P11. |
|  | 13c | V | For validation, show a comparison with the development data of the distribution of important variables (demographics, predictors and outcome) | Table 1 P26 |
| Model development | 14a | D | Specify the number of participants and outcome events in each analysis. | P26 footnote to Table 1 |
|  | 14b | D | If done, report the unadjusted association between each candidate predictor and outcome. | P28 Table 2. Univariable estimates in Tables S5 P12 and S3 P9. |
| Model specification | 15a | D | Present the full prediction model to allow predictions for individuals (i.e., all regression coefficients, and model intercept or baseline survival at a given time point). | P28 Table 2 plus log odds estimates and intercept in Table S7. |
|  | 15b | D | Explain how to the use the prediction model. | P13 and Figure 1 on P30 |
| Model performance | 16 | D, V | Report performance measures (with CIs) for the prediction model. | P12-13. We use interquartile range as an indication of range given the use of multiple imputation to handle missing data |
| Model updating | 17 | V | If done, report the results from any model updating (i.e. model specification, model performance) | P10 describes re-calibration updates implemented according to previous recommendations. P13 in results. |
| Discussion |  |  |  |  |
| Limitations | 18 | D, V | Discuss any limitations of the study (such as nonrepresentative sample, few events per predictor, missing data). | P16 |
| Interpretation | 19a | V | For validation, discuss the results with reference to performance in the development data, and any other validation data. | P12-13 |
|  | 19b | D, V | Give an overall interpretation of the results, considering objectives, limitations, and results from similar studies, and other relevant evidence. | P15-18 |
| Implications | 20 | D, V | Discuss the potential clinical use of the model and implications for future research. | P17-18 |
| Other information |  |  |  |  |
| Supplementary information | 21 | D, V | Provide information about the availability of supplementary resources, such as study protocol, Web calculator, and data sets. | P6 notes Supporting Information and access to ALSPAC data |
| Funding | 22 | D, V | Give the source of funding and the role of the funders for the present study. | P20 |

Items relevant only to the development of a prediction model are denoted by D, items relating solely to a validation of a prediction model are denoted by V, and items relating to both are denoted D,V.

**Appendix S6. Public and patient involvement work**

Our public and patient involvement work on this project involved a focus group via videoconferencing with four young people who either had lived experience of anxiety and depression or had a parent with recurrent depression. The group discussed a research project about developing a depression risk prediction tool. One researcher (RBJ) led the facilitation of the discussion and was supported by AS and one other team member. There were mixed feelings about the tool, with participants noting both potential benefits and concerns. Potential advantages raised were that the tool could be useful for prevention, that it might encourage some individuals identified by the tool as potentially being vulnerable to do their own research or to get some extra information or education on the topic. The research that informed the prediction tool was also identified as important because that can inform widespread practice and policy. Potential disadvantages mainly related to ensuring that the development and implementation of such a prediction tool in practice were done in a humane, non-stigmatising way and acknowledged the complexity of depression. For instance, one young person expressed concerns that if a prediction tool “labelled” a young person as at risk, this might create a “self-fulfilling prophecy” - where a young person could be told they are “susceptible to depression” and following a stressor, this could send them “into a spiral”. On a similar theme, another view was it could be “dehumanising” if they are told that they have a certain “characteristic or experience”, and “you might end up defining yourself by that or it might be something you can’t change”. The importance of ensuring such a tool did not widen inequalities and access to care was also noted. There was also a discussion about the need for this tool to be implemented with clinical support, for example “in a surgery or dedicated mental health centre” to ensure immediate access to help was available, supporting the use in primary care.

References

Angold, A., & Costello, E. J. (1995). A Test—Retest Reliability Study of Child-Reported Psychiatric Symptoms and Diagnoses using the Child and Adolescent Psychiatric Assessment (CAPA-C). *Psychological Medicine*, *25*(4), 755–762. [https://doi.org/10.1017/S0033291700034991](about:blank)

Avenevoli, S., Swendsen, J., He, JP., Burstein, M., Merikangas, K.R., (2015). Major depression in the national comorbidity survey-adolescent supplement: prevalence, correlates, and treatment. J Am Acad Child Adolesc Psychiatry, 54(1):37-44.e2.

Belli, R. F. (1998). The Structure of Autobiographical Memory and the Event History Calendar: Potential Improvements in the Quality of Retrospective Reports in Surveys. *Memory*, *6*(4), 383–406. [https://doi.org/10.1080/741942610](about:blank)

Birmaher, B., Brent, D.A., Chiappetta, L., Bridge, J., Monga, S., & Baugher, M. (1999). Psychometric Properties of the Screen for Child Anxiety Related Emotional Disorders (SCARED): A Replication Study. *Journal of the American Academy of Child & Adolescent Psychiatry*, *38*(10), 1230–1236. [https://doi.org/10.1097/00004583-199910000-00011](about:blank)

Costello, E. J., & Angold, A. (1988). Scales to assess child and adolescent depression: checklists, screens, and nets. Journal of the American Academy of Child & Adolescent Psychiatry, 27(6), 726-737.

Cox, J. L., Holden, J. M., & Sagovsky, R. (1987). Detection of Postnatal Depression: Development of the 10-item Edinburgh Postnatal Depression scale. *British Journal of Psychiatry*, *150*(JUNE), 782–786. [https://doi.org/10.1192/bjp.150.6.782](about:blank)

Gerdin, M., Roy, N., Khajanchi, M., Kumar, V., Felländer-Tsai, L., Petzold, M., Tomson, G., von Schreeb, J., Amit, G., Ashish, J., Debojit, B., Deen, M. I., Dusu, Y., Jegadeesa, K., Jyoti, K., Makhan, L. S., Mangesh, N., Ranganathan, J., Samarendra, N. G., … Veera, K. (2016). Validation of a novel prediction model for early mortality in adult trauma patients in three public university hospitals in urban India. *BMC Emergency Medicine*, *16*(1), 1–12. [https://doi.org/10.1186/s12873-016-0079-0](about:blank)

Gest, S. D., Reed, M. J., & Masten, A. S. (1999). Measuring developmental changes in exposure to adversity: A life chart and rating scale approach. *Development and Psychopathology*, *11*, 171–192.

Gunlicks, M.L., & Weissman, M.M. (2008). Change in child psychopathology with improvement in parental depression: a systematic review. *J Am Acad Child Adolesc Psychiatry,* 47(4):379-389.  doi: 10.1097/CHI.0b013e3181640805.

Hall, R. C., & Parks, J. (1995). The Modified Global Assessment of Functioning Scale: Addendum. *Psychosomatics*, *36*(4), 416–417. https://doi.org/10.1016/s0033-3182(95)71656-5

Johnson, J. H., & McCutcheon , S. (1980). Assessing Life Stress in Older Children and Adolescents: Preliminary Findings With the Life Events Checklist. *Hemisphere Publishing Corp.*

Kroenke, K., Spitzer, R. L., & Williams, J. B. W. (2001). The PHQ-9: Validity of a brief depression severity measure. *Journal of General Internal Medicine*, *16*(9), 606–613. [https://doi.org/10.1046/j.1525-1497.2001.016009606.x](about:blank)

Rice, F., Sellers, R., Hammerton, G., Eyre, O., Bevan-Jones, R., Thapar, A. K., Collishaw, S., Harold, G. T., & Thapar, A. (2017). Antecedents of new-onset major depressive disorder in children and adolescents at high familial risk. *JAMA Psychiatry*, *74*(2), 153–160. [https://doi.org/10.1001/jamapsychiatry.2016.3140](about:blank)

Ridley M, Rao G, Schilbach F, Patel V. (2000). Poverty, depression, and anxiety: Causal evidence and mechanisms. Science,370(6522):eaay0214. doi: 10.1126/science.aay0214.

Steyerberg, E. W., & Vergouwe, Y. (2014). Towards better clinical prediction models: Seven steps for development and an ABCD for validation. *European Heart Journal*, *35*(29), 1925–1931. [https://doi.org/10.1093/eurheartj/ehu207](about:blank)

Thabrew, H., Stasiak, K., Bavin, L. M., Frampton, C., & Merry, S. (2018). Validation of the Mood and Feelings Questionnaire (MFQ) and Short Mood and Feelings Questionnaire (SMFQ) in New Zealand help-seeking adolescents. *International Journal of Methods in Psychiatric Research*, *27*(3), 1–9.

Van Calster, B., Van Hoorde, K., Valentin, L., Testa, A. C., Fischerova, D., Van Holsbeke, C., Savelli, L., Franchi, D., Epstein, E., Kaijser, J., Van Belle, V., Czekierdowski, A., Guerriero, S., Fruscio, R., Lanzani, C., Scala, F., Bourne, T., Timmerman, D., & International Ovarian Tumour Analysis Group (2014). Evaluating the risk of ovarian cancer before surgery using the ADNEX model to differentiate between benign, borderline, early and advanced stage invasive, and secondary metastatic tumours: prospective multicentre diagnostic study. *BMJ (Clinical research ed.)*, *349*, g5920. [https://doi.org/10.1136/bmj.g5920](about:blank)

Wickramaratne, P., Gameroff, M.J., Pilowsky, D.J., Hughes, C.W., Garber, J., Malloy, E., King, C., Cerda, G., Sood, A.B., Alpert, J.E., Trivedi, M.H., Fava, M., Rush, A.J., Wisniewski, S., Weissman, M.M. (2011). Children of depressed mothers 1 year after remission of maternal depression: findings from the STAR*D-Child study. *Am J Psychiatry,168(6),* 593-602.

Wing, J. K., Babor, T., Brugha, T., Burke, J., Cooper, J. E., Giel, R., Jablenski, A., Regier, D., & Sartorius, N. (1990). SCAN. Schedules for Clinical Assessment in Neuropsychiatry. *Archives of general psychiatry*, *47*(6), 589–593. https://doi.org/10.1001/archpsyc.1990.01810180089012
